# Epidemiological and molecular analysis of human norovirus infections in Taiwan during 2011 and 2012

**DOI:** 10.1186/1471-2334-13-338

**Published:** 2013-07-22

**Authors:** Meng-Bin Tang, Chien-Hsien Chen, Shou-Chien Chen, Yu-Ching Chou, Chia-Peng Yu

**Affiliations:** 1 Department of Family Medicine, Wei-Gong Memorial Hospital, Toufen Township, Miaoli County, Taiwan; 2 Department of Bioengineering, Tatung University, Taipei, Taiwan; 3 General Education Center, Tatung University, Taipei, Taiwan; 4 Department of Family Medicine, Da-Chien General Hospital, Miaoli City, Miaoli County, Taiwan; 5 School of Public Health, National Defense Medical Center, Taipei, Taiwan

**Keywords:** Human norovirus, Molecular epidemiology, RT-PCR, Taiwan

## Abstract

**Background:**

The human norovirus (NV) circulates worldwide and is a major cause of epidemics, which have increased in Taiwan since 2002. NV in acute gastroenteritis (AGE) and non-acute gastroenteritis (asymptomatic) patients, including children and adults, have not been previously examined in Taiwan; therefore, we examined the epidemiology and phylogeny of NV in AGE and asymptomatic patients of all ages.

**Methods:**

253 stool samples were collected from August 2011 to July 2012 (including 155 AGE and 98 asymptomatic samples in Taiwan) and analyzed using reverse transcription-polymerase chain reaction (RT-PCR) for NV. Primers targeting the RNA-polymerase gene were used for RT-PCR to allow DNA sequencing of Taiwan NV strains and phylogenetic analyses.

**Results:**

NV was detected in 24 (9.5%) of 253 stool specimens using RT-PCR. NV was isolated from all age groups (1 to 86 y) and those NV-positive samples were major identified from inpatients (79.2%, 19/24). Statistical analysis showed that the NV infectious rate of AGE patients was statistically significant (*P* < 0.05) for age, season and water type, respectively. Phylogenetic analyses of the RdRp region sequence showed that 24 NV isolates belonged to Genogroup II Genotype 4 (GII.4). They were closely related to the epidemic strain in Taiwan in 2006, the GII.4-2006b pandemic strain in 2006, and the GII.4-New Orleans strain in 2010.

**Conclusion:**

This study is the first to examine NV in sporadic AGE and asymptomatic patients in Taiwan. Furthermore, epidemic strains of isolated GII.4 were predominant in Taiwan during 2011 and 2012.

## Background

Viral gastroenteritis is a major threat with high morbidity and mortality, especially in children, elderly people, and immunocompromised people in developing and developed countries. It is estimated that viral gastroenteritis is the cause of 30%-40% of infectious cases in developed countries [1]. Human noroviruses (NV) are the leading cause of epidemic acute non-bacterial gastroenteritis worldwide. Taiwan has experienced a substantial increase in NV outbreaks and sporadic records since 2002 [2]. Enzyme-linked immunosorbent assays showed that 20% of pediatric viral gastroenteritis cases were caused by NV from 2004 to 2005 [3]. Four NV outbreaks occurred in a psychiatric care center, and the mean incidence rate of NV gastroenteritis in hospitalized patients during these outbreaks was 12.7% from 2005 to 2007 [4].

NV belongs to the *Caliciviridae* family and has a single-stranded RNA genome of 7.5-7.7 kb. They are currently classified into 5 genogroups (GI to GV) [5], and only NV GI, GII, and GIV have been associated with human gastroenteritis [6]. NV is spread through a number of pathways with the occurrence of both fecal-oral and vomit-oral transmission. Direct person-to-person transmission is a primary mode of transmission in most outbreaks [7] and sporadic diseases [8]. Furthermore, NV disease outbreaks are reported year-round. They peak during months with cold weather and temperate climates [9].

NV sporadic cases involving children have been reported in Taiwan [10-13]. However, data on the molecular epidemiology (including epidemic genotyping, ages, and seasonality) of NV infection in acute gastroenteritis (AGE) and non-acute gastroenteritis (asymptomatic) patients in Taiwan are limited. The objectives of the study were as follows: to determine the AGE and asymptomatic infection rates of NV among hospital patients; to examine the association of patients age and of infection seasons with the NV infection rates; to analyze the NV genotypes by RT-PCR and sequencing methods.

## Methods

### Case definition

AGE patients were defined as patients with clinical diarrhea (≧3 loose stools within a 24 h period), which may be accompanied by abdominal pain, fever, nausea, and vomiting. Asymptomatic patients were defined as patients undergoing routine medical examinations without symptoms of clinical diarrhea.

### Specimen collection

This study was approved by the Human Subject Research Ethics Committee of the Wei-Gong Memorial Hospital and the approval number was 100003. Informed written consent was obtained from adult participants and parents of minors. This study was conducted from August 2011 to July 2012 at Wei-Gong Memorial Hospital in Taiwan. The stools of 253 patients (155 AGE and 98 asymptomatic patients) were collected. The stool samples were stored at -20°C before transfer on ice blocks to the Department of Bioengineering, Tatung University, where they were stored at -70°C. The samples were frozen (-70°C) before or after initial analysis as fecal suspensions. The samples were examined for the presence of NV using RT-PCR before storage in a balanced salt solution at 10% suspensions at -70°C until use.

### Nucleic acid extraction and RT-PCR

Nucleic acid was extracted using a viral nucleic acid extraction kit (Geneaid, Taiwan) from 200 μL of 10% fecal suspension to a final volume of 50 μL of RNase-free H_2_0. RT-PCR for NV was performed using 10 μL nucleic acid with 10 μL of RT-PCR mix (Qiagen, Taiwan) containing the RT-PCR mix for NV contained 0.5 μL (10 μM) of JV12 (5’ATACCACTATGATGCAGATTA-3’, nucleotides location 4552–4572) and JV13 (5’-TCATCATCACCATAGAAAGAG-3’, nucleotides location 4878–4858) primers [14], a 4 μL buffer, 0.4 μL (10 mM) dNTPs, 3.8 μL H_2_O, and 0.8 μL (1.25 U/μL) of enzyme mix. The thermal conditions for NV-specific one-step RT-PCR were 50°C for 30 min and 95°C for 15 min, 40 cycles at 94°C for 30 s, 37°C for 1 min, and 72°C for 1 min, followed by a final extension of 72°C for 10 min. The amplicons were analyzed in 2% agarose gel electrophoresis at 100 V for 30 min and visualized under UV light after ethidium bromide staining. Positive PCR products were stored at -20°C. All NV positive samples were subjected to sequence and phylogenetic analyses.

### Sequence and phylogenetic analyses

NVs were identified based on nucleotide sequences of the RNA-dependent RNA polymerase (RdRp) region, which comprised 327 bp for the positive control. All NV PCR product sequences were analyzed using the basic local alignment search tool (BLAST) and DNAMAN software. Phylogenetic trees with 1000 bootstrap replicates were generated using the neighbor-joining method by employing molecular evolutionary genetics analysis (MEGA), version 5.0. Reference strains were downloaded from the GenBank. Only bootstrap values >65 were considered significant.

### Statistical analysis

For categorical variables, the chi-square test was used to examine differences in proportions between groups. *P* values < 0.05 were considered statistically significant. Fisher’s exact tests were used when the expected value for a cell was <5.

## Results

### Study population

During the study period, 253 patients (including children and adults) comprising 136 (53.8%) males were enrolled. Among them, 155 (61.3%) were AGE patients. The samples (including 53 obtained from outpatients, 6 from emergency unit, and 194 from inpatients) were collected and screened for NV. All sample characteristics by the enrolment site are shown in Table 1.

**Table 1:** Epidemiological and clinical features by examine NV in AGE, asymptomatic patients

	**AGE patients (n = 155)**					**Asymptomatic patients (n = 98)**		
	**NV**	**Negative**				**NV**	**Negative**	
**Parameter**	**(n = 17)**	**(n = 138)**	***P***^**a**^	**OR**^**d**^	**95% CI**^**d**^	**(n = 7)**	**(n = 91)**	***P***^**b**^
Detection rate (%)	**11**	**-**				**7.1**	**-**	
Sex (male: female)	**10:7**	**74:64**				**5:2**	**47:44**	
Setting								
Outpatient (38,15) ^**c**^	**2(11.8)**	**36(26.1)**	**-**	**-**	**-**	**3(42.8)**	**12(13.2)**	**-**
Emergency (6,0) ^**c**^	**-**	**6(4.3)**	**-**	**-**	**-**	**-**	**-**	**-**
Inpatient (111,83) ^**c**^	**15(88.2)**	**96(69.6)**	**-**	**-**	**-**	**4(57.2)**	**79(86.8)**	**-**
Age								
<10	**9(52.9)**	**23(16.7)**	**0.002**	**5.6**	**2.0-16.1**	**1(14.3)**	**4(4.4)**	**-**
10-40	**3(17.7)**	**28(20.3)**	**-**	**-**		**2(28.6)**	**5(5.5)**	**-**
>40	**5(29.4)**	**87(63.0)**	**0.016**	**0.2**	**0.1-0.7**	**4(57.1)**	**82(90.1)**	**-**
Season								
Spring	**3(17.6)**	**54(39.1)**	**-**	**-**	**-**	**-**	**23(25.3)**	**-**
Summer	**2(11.8)**	**42(30.4)**	**-**	**-**	**-**	**4(57.1)**	**45(49.4)**	**-**
Fall	**2(11.8)**	**10(7.3)**	**-**	**-**	**-**	**-**	**10(11.0)**	**-**
Winter	**10(58.8)**	**32(23.2)**	**0.004**	**4.7**	**1.7-13.4**	**3(42.9)**	**13(14.3)**	**-**
Water type								
Tap water	**12(70.6)**	**118(85.5)**	**-**	**-**	**-**	**6(85.7)**	**81(89.0)**	**-**
Underground water	**-**	**2(1.4)**	**-**	**-**	**-**	**-**	**-**	**-**
Spring water	**4(23.5)**	**7(5.1)**	**0.022**	**5.8**	**1.5-22.3**	**-**	**2(2.2)**	**-**
Miss data	**1(5.9)**	**11(8.0)**	**-**	**-**	**-**	**1(14.3)**	**8(8.8)**	**-**
Fever >38°C (%)				**-**	**-**			
Yes	**9(52.9)**	**47(34.1)**	**-**	**2.5**	**0.9-7.1**	**2(28.6)**	**21(23.1)**	**-**
No	**7(41.2)**	**91(65.9)**	**-**	**-**	**-**	**5(71.4)**	**70(76.9)**	**-**
Miss data	**1(5.9)**	**-**	**-**	**-**	**-**	**-**	**-**	**-**
Vomiting								
Yes	**7(41.2)**	**28(20.3)**	**-**	**2.7**	**0.9-7.7**	**-**	**6(6.6)**	**-**
No	**10(58.8)**	**108(78.3)**	**-**	**-**	**-**	**7(100)**	**85(93.4)**	**-**
Miss data	**-**	**2(1.4)**	**-**	**-**	**-**	**-**	**-**	**-**
Stool type								
Watery	**8(47.1)**	**49(35.5)**	**-**	**-**	**-**	**-**	**1(1.1)**	**-**
Bloody	**1(5.8)**	**18(13.0)**	**-**	**-**	**-**	**-**	**3(3.3)**	**-**
Non-watery, non-bloody	**8(47.1)**	**71(51.5)**	**-**	**-**	**-**	**7(100)**	**87(95.6)**	**-**
Abdominal pain								
Yes	**9(52.9)**	**84(60.9)**	**-**	**-**	**-**	**-**	**19(20.9)**	**-**
No	**7(41.2)**	**52(37.7)**	**-**	**-**	**-**	**7(100)**	**72(79.1)**	**-**
Miss data	**1(5.9)**	**2(1.4)**	**-**	**-**	**-**	**-**	**-**	**-**

Only significant *p* value (<0.05) reported.

^a^ two-tailed Chi-Square, Fisher’s exact test comparing AGE patient and negative cases.

^b^ two-tailed Chi-Square, Fisher’s exact test comparing asymptomatic patient and negative cases.

^c^ the table in parentheses refer to the former is AGE patients and the latter is asymptomatic patients number.

^d^*OR* odds ratio, *CI* confidence interval.

### NV positive rates and clinical features

NV was detected in 24 (9.5%) of all samples, 17 (11%) in AGE patients, and 7 (7.1%) in asymptomatic patients. The clinical feature of NV that detected in AGE and asymptomatic patients, were fever, vomiting, and abdominal pain (Table 1). AGE patients with NV-positive were significantly higher in fever and in vomiting than with NV-negative (Table 1). Clinical symptoms of NV-positive AGE include fever (61.1%, 11/18), vomiting (42.1%, 8/19), watery stool (42.1%, 8/19), and abdominal pain (50%, 9/18) (Table 2).

**Table 2:** Characteristics of NV-associated with AGE and asymptomatic patients

**No.**	**Years**	**Location**	**Genotype**	**AGE**	**Fever**	**Vomiting**	**Stool**	**Abdominal pain**
**2**	13	Inpatient	GII.4	Y^a^	Y	Y	Nn^b^	Miss
**10**	5	Inpatient	GII.4	Y	Y	N	Nn	N
**14**	40	Inpatient	GII.4	N^a^	N	N	Nn	N
**22**	22	Outpatient	GII.4	Y	N	N	Watery	N
**27**	7	Inpatient	GII.4	Y	Y	Y	Watery	Y
**31**	1	Inpatient	GII.4	Y	Y	N	Nn	N
**37**	46	Outpatient	GII.4	N	N	N	Nn	N
**40**	6	Inpatient	GII.4	Y	Y	Y	Watery	Y
**41**	31	Outpatient	GII.4	N	N	N	Nn	N
**44**	50	Inpatient	GII.4	Y	N	N	Watery	Y
**45**	2	Inpatient	GII.4	Y	Y	N	Watery	N
**49**	86	Inpatient	GII .4	Y	Y	N	Nn	Y
**51**	68	Inpatient	GII.4	Y	N	N	Nn	Y
**54**	17	Outpatient	GII.4	Y	N	Y	Nn	N
**56**	1	Inpatient	GII.4	Y	Y	N	Watery	N
**84**	71	Inpatient	GII .4	Y	Miss	N	Nn	N
**107**	2	Inpatient	GII.4	Y	N	Y	Watery	Y
**122**	2	Inpatient	GII.4	Y	Y	Y	Watery	Y
**171**	1	Inpatient	GII.4	Y	N	Y	Nn	Y
**183**	75	Inpatient	GII.4	N	Y	N	Nn	N
**184**	82	Inpatient	GII.4	Y	N	Y	Bloody	Y
**191**	66	Inpatient	GII.4	N	N	N	Nn	N
**193**	1	Inpatient	GII.4	N	Y	N	Nn	N
**203**	75	Outpatient	GII.4	N	N	N	Nn	N

a:*Y* Yes, *N* No.

b:*Nn* Non-watery, non-bloody stool.

### Epidemiological features

**Figure 1:**
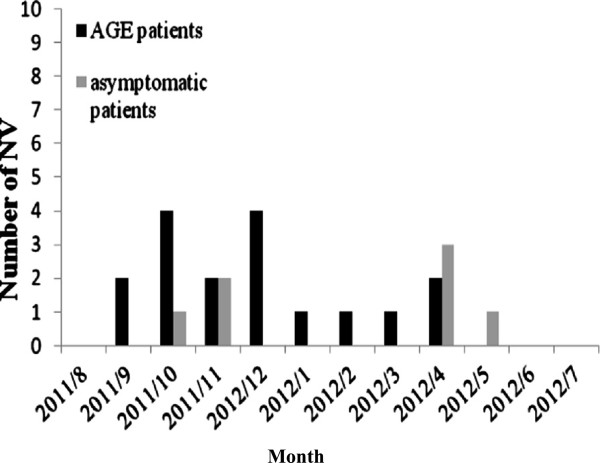
Monthly distribution of NV-associated with in AGE and asymptomatic patients.

**Figure 2:**
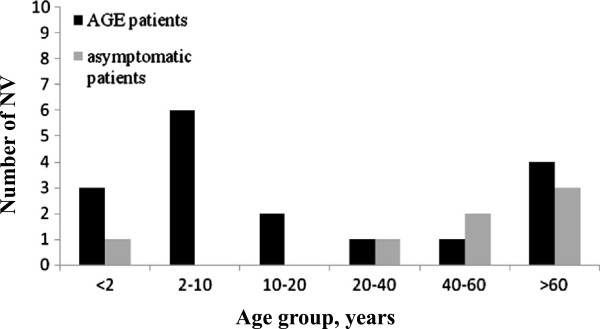
Age group distribution of NV-associated with AGE and asymptomatic patients.

AGE patients with NV-positive were more likely to drink spring water than NV-negative patients (23.5%, 4/17 versus 5.1%, 7/138) (Table 1). NV infections were identified in AGE patients of all ages (1 to 86 y) (Table 2). NV was detected more often in AGE patients less than 10 years old (52.9%, 9/17 versus 16.7%, 23/138), and those greater than 40 years old (29.4%, 5/17 versus 63%, 87/138) (Table 1 and Figure 1). However, asymptomatic patients with NV-positive were identified both from <2 and from >20 years old. Those NV-positive samples were major identified from inpatients (79.2%, 19/24) (Table 2). NV infections were identified throughout the year (highest incidence in December, followed by October) among patients and controls. NV was detected in AGE patients more often in the winter than in the other seasons (Table 1 and Figure 2).

### Phylogenetic analyses

**Figure 3:**
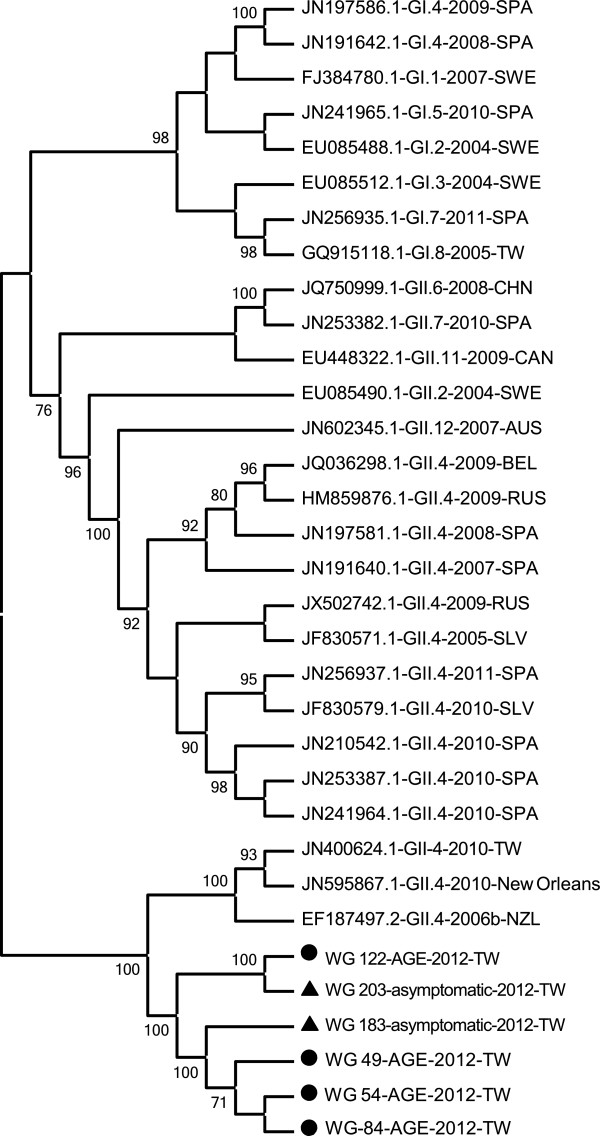
**Phylogenetic tree based on nucleotide sequence of the NV RdRp region, the reference strain designation is the Genbank access number followed by the genotype designation.** In this study, the isolated strain designation is the Wei-Gong (WG) hospital sample number.

Twenty-four NVs that were identified and sequenced belonged to the NV Genogroup II.4 (GII.4). Three GII.4 variants (GII.4-2006b, GII.4-2010tw, and GII.4-New Orleans variants) were identified, as shown in Figure 3.

## Discussion

NVs are the leading cause of AGE among people [15]. NV is associated with 6%-25% of AGE patients worldwide [16-20]. Previous studies have indicated that the NV positive rate differs significantly between AGE (16.1%) and asymptomatic (5.2%) [21]. The NV positive rate (11%) among AGE patients in this study was similar to those in Taiwan (10.5%) [22] and Australia (11.4%) [23]. In this study, the NV positive detection rate although was so low, but its prevalence rate was in accordance with other reports about the recent Taiwan situation (2007 prevalence of 8.2%, 2010 prevalence of 14.6%) [10,12]. The major reason to explain why NV positive rate so low was the lower gastroenteritis fecal samples. So we will continue to collect more gastroenteritis fecal samples in the next year. Furthermore, the NV positive rate (7.1%) among asymptomatic patients was similar to that in India (7.5%) [24]. Therefore, this study shows that the predominance of NV infections in Taiwan is a pandemic and may indicate the circulation of this strain in this region.

A previous study indicated that GII.4 strains of NV were dominant in AGE and asymptomatic patients during a recent hospital outbreak [25]. This result is similar to that obtained in this study. The GII.4 strains are a major cause of sporadic occurrences of AGE. Although NV GII.4 strains are widespread, they do not always cause AGE, which may account for the increased number of infections through asymptomatic transmission routes [26]. Furthermore, the population usually includes asymptomatic carriers of NV related to the occurrence of NV outbreaks [27]. Therefore, commonly occurring NV infections may result from multiple routes of transmission, especially for asymptomatic patients with NV pathways.

A previous study [28] indicated that the NV positive rate of AGE patients differs significantly between inpatients (16.7%) and outpatients (9.2%). The NV positive rate for AGE and asymptomatic patients that from inpatients and outpatients (Table 1) was similar to that of recent studies [21,24,29-35]. Therefore, in this study, the result appeared that AGE patients with NV-positive from inpatients were higher than from outpatients.

Among all NV GII.4 samples in this study, those samples were inpatients (79.2%) and outpatients (20.8%), respectively. Similar to a previous study [36], the analysis indicated that severe outcomes are associated with NV GII.4 infections. Therefore, epidemic strains of isolated GII.4 may cause severe clinical symptoms, and patients may require hospitalization.

NV in AGE patients was significantly associated with the consumption of spring water [37]. Previous studies are consistent with our observation that AGE-related NV infections are significantly related to the consumption of spring water. Similar to a previous study, the analysis indicated that the GII strain was associated with spring water (OR = 3.8). The consumption of spring water is a risk factor and suspected transmission route. A previous study indicated that NV in AGE patients was significantly associated with clinical symptoms [38]. This was similar to our observation that AGE-related NV infections have marginal statistical significance related to the diagnosis of fever and vomiting. The analysis also indicated that the GII strain was associated with vomiting. Both fever and vomiting are risk factors and may be used by clinicians for the clinical diagnosis of acute gastroenteritis in patients.

NV is a leading cause of sporadic gastroenteritis in children and adults. It is a common cause of hospitalization for gastroenteritis, especially among vulnerable populations (young children and elderly people). Similar to the results of previous studies [39], AGE-related NV infections have statistical significance in patients aged <10 and >40 years old compared to other age groups. It indicated that young children and elderly people may be at risk of NV infection. In this study, patients aged 1 to 86 years were infected with NV, indicating NV infections in all age groups. This indicated the predominance of NV infections in young children and elderly people, and may provide health authorities in Taiwan with control strategies to prevent person-to-person NV transmission.

Transmission of NV infections occurred year-round in most surveys, and a cold weather peak was observed in 11 of the 12 studies [9]. The detection rate of AGE-related NV infections is significantly greater during the winter compared to the other seasons [40,41]. This epidemiologic feature of NV has crucial implications regarding the mode of transmission and understanding the etiology of gastroenteritis in children and adults.

Human diseases are primarily caused by GI and GII NVs, and most outbreaks are caused by GII.4 strains [42]. The major pandemic genotype of NV in the world was GII.4, also include Taiwan [2,4]. New GII.4 strains have emerged every 2–3 y over the past decade, and have replaced previously predominant GII.4 strains [43,44]. In the previous study, it also indicated that NV GII was prevalent in some area only, without GI [45,46]. The similar result was appeared in this study. Furthermore, it had ever been proved that the major cause of one gastroenteritis outbreak in Taiwan was NV GII.4 (100%, 31/31) only [47]. So we believed that only NV GII.4 did prevalent in the gastroenteritis patients in this study. NV GII.4-2006b was associated with sporadic gastroenteritis and was a globally circulating strain in several countries, including Japan, China, and Italy [48-50]. From previous reports [48,49] and our result, it proved that AGE patients infected with NV GII.4-2006b could be more prevalent in younger children and in winter season. The total GII.4 isolate strains were closely related to the epidemic strain in Taiwan in 2006, the GII.4-2006b pandemic strain in 2006, and the strain in New Orleans (United States) in 2010. It is crucial to emphasize the existence of 2 variants of GII.4 (GII.4-2006b and GII.4-New Orleans) in Taiwan. NV GII.4 variant strains implicated in sporadic gastroenteritis worldwide also occurred in Taiwan, indicating global spread.

## Conclusion

In summary, this study is the first to examine NV in sporadic AGE and asymptomatic patients in Taiwan. The results showed that the season and age distribution for NV-positive patients were similar to those in previous NV studies worldwide. Furthermore, our results indicated that epidemic strains of isolated GII.4 were predominant in Taiwan during 2011 and 2012. Systematic surveillance and evidence-based studies are required to determine the transmission pathways and spread of NV.

## Competing interests

The authors declare that they have no competing interests.

## Authors’ contributions

MBT, SCC designed the epidemiological study, participated in its design and coordination. YCC performed the statistical analysis and participated in its design. SCC conceived of the study, and participated in its design and coordination and helped to draft the manuscript. CPY performed the laboratory test on human stool samples, carried out the molecular studies, participated in the sequence alignment and drafted and edited the manuscript. All authors read and approved the final manuscript.

## Pre-publication history

The pre-publication history for this paper can be accessed here:

http://www.biomedcentral.com/1471-2334/13/338/prepub
